# Progress in Additive Manufacturing of Magnesium Alloys: A Review

**DOI:** 10.3390/ma17153851

**Published:** 2024-08-03

**Authors:** Jiayu Chen, Bin Chen

**Affiliations:** School of Materials Science and Engineering, Shanghai Jiao Tong University, Shanghai 200240, China; hebery28@sjtu.edu.cn

**Keywords:** additive manufacture, magnesium alloy, selective laser melting, wire arc additive manufacturing, jetting technologies, friction stir additive manufacturing, indirect additive manufacturing

## Abstract

Magnesium alloys, renowned for their lightweight yet high-strength characteristics, with exceptional mechanical properties, are highly coveted for numerous applications. The emergence of magnesium alloy additive manufacturing (Mg AM) has further propelled their popularity, offering advantages such as unparalleled precision, swift production rates, enhanced design freedom, and optimized material utilization. This technology holds immense potential in fabricating intricate geometries, complex internal structures, and performance-tailored microstructures, enabling groundbreaking applications. In this paper, we delve into the core processes and pivotal influencing factors of the current techniques employed in Mg AM, including selective laser melting (SLM), electron beam melting (EBM), wire arc additive manufacturing (WAAM), binder jetting (BJ), friction stir additive manufacturing (FSAM), and indirect additive manufacturing (I-AM). Laser powder bed fusion (LPBF) excels in precision but is limited by a low deposition rate and chamber size; WAAM offers cost-effectiveness, high efficiency, and scalability for large components; BJ enables precise material deposition for customized parts with environmental benefits; FSAM achieves fine grain sizes, low defect rates, and potential for precision products; and I-AM boasts a high build rate and industrial adaptability but is less studied recently. This paper attempts to explore the possibilities and challenges for future research in AM. Among them, two issues are how to mix different AM applications and how to use the integration of Internet technologies, machine learning, and process modeling with AM, which are innovative breakthroughs in AM.

## 1. Introduction

As the lightest structural metal with abundant resources, a magnesium alloy is crucial in replacing traditional steel materials and achieving substantial weight reduction across various applications, including missiles and satellites. Furthermore, magnesium aligns with the global trend toward green and low-carbon development, which has led to increased interest in recent years [1,2].

**Figure 1:**
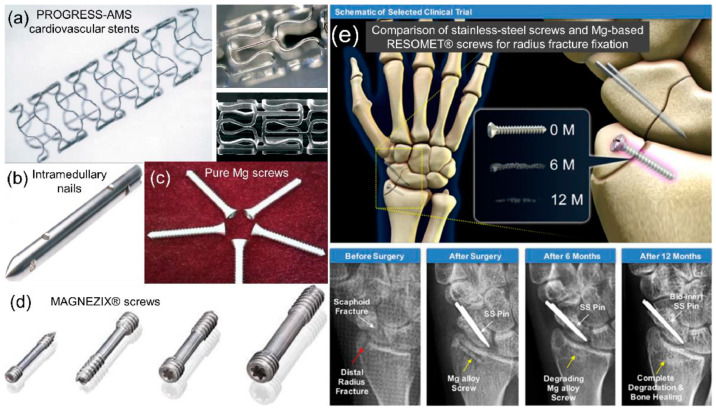
Examples of Mg alloy biomedical implants employed in clinical applications [13].

Magnesium exhibits several outstanding characteristics, such as its ease of cutting, high specific strength, high damping capacity, low melting point, and excellent casting ability [3,4]. Due to these advantages, magnesium alloys find applications in various fields, including automotive, consumer electronics, aerospace, and biomedical engineering. Specifically, the elastic modulus and yield strength of Mg alloys are comparable to those of human bone. Moreover, compared to aluminum alloy, titanium, and steel, Mg has the lowest engineering density at only 1.74 g/cm^3^ [5,6]. These qualities have made magnesium a candidate for medical applications (Figure 1), such as orthopedics, maxillofacial procedures, and cardiology [7]. However, the brittleness of magnesium, attributable to its inherent hexagonal close-packed (HCP) crystal structure, limits its use in manufacturing large and complex structural components [8,9,10,11,12].

**Figure 2:**
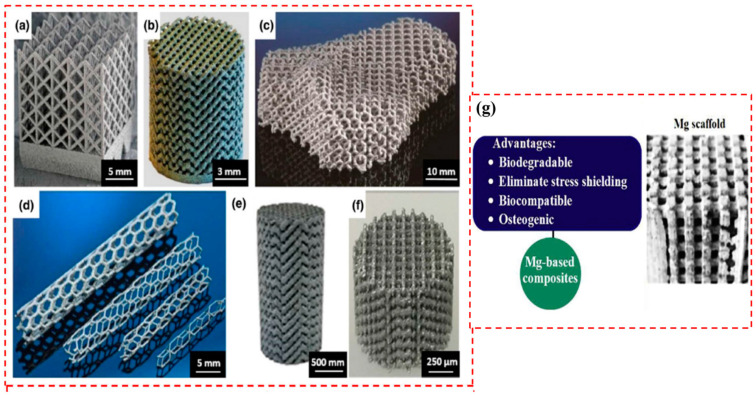
(**a**–**f**) Mg alloy scaffolds manufactured using LPBF process. (**g**) biomedical components of LPBFed Mg alloy and their application [24].

Additive manufacturing differs from subtractive manufacturing techniques, such as machining, in that objects are created layer by layer from 3D model data by adding materials [14]. Deformation machining is challenging for processing complex shapes, and the casting process often introduces defects, such as impurity inclusion, coarse grains, and shrinkage, which ultimately affect the mechanical properties of the final parts [15,16,17,18]. According to research [19,20], traditional manufacturing techniques like die-casting and hot extrusion for magnesium alloys often result in components with inadequate mechanical strength and permeability. In comparison, additive manufacturing emerges as a superior alternative, surpassing both conventional deformation and casting processes in terms of product quality and mechanical performance. Additive manufacturing offers significant advantages, including an easy process, improved pore structure, reduced corrosion, and precise construction of internal structures. It also refines grain and optimizes microstructures [21,22]. Additionally, AM overcomes the limitations of traditional manufacturing routes, such as constructive and subtractive manufacturing, and can precisely manufacture complex internal and external geometries, thus expanding the range of design possibilities (Figure 2) [23].

The inherent defects of the matrix magnesium alloy will pose challenges to the additive manufacturing process, including issues like cracks, inclusions, and low vaporization temperature [1]. Furthermore, the typical forms of the additive manufacturing of raw materials are powder, wire, and liquid resin. This form increases the surface energy of the metal, thereby elevating the risk of reacting with atmospheric oxygen. To mitigate the effects of oxidation, specialized airtight equipment capable of printing magnesium in an inert atmosphere is necessary. However, due to the stringent safety requirements of the equipment, the research on the manufacturing process of magnesium is somewhat inadequate [25,26].

**Figure 3:**
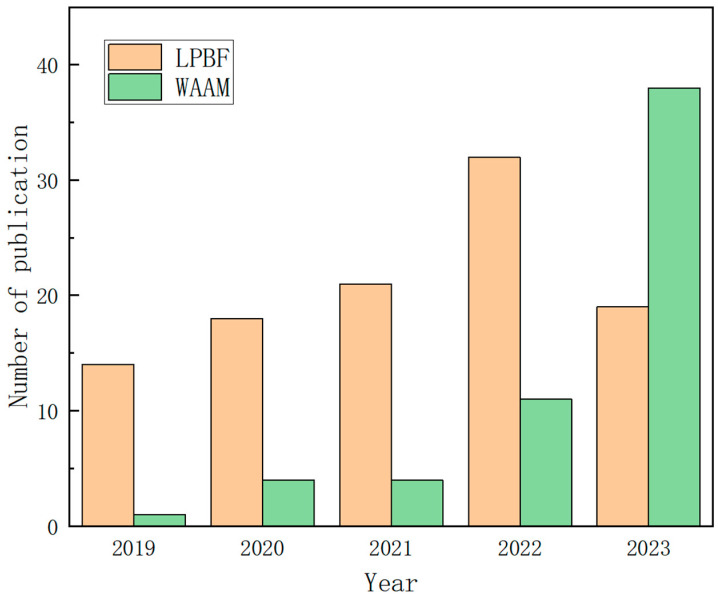
Publication trends of AMed Mg alloy comparison showing publication trends of LPBF and WAAM of Mg alloys year-wise (WAAM&Mg or WAAM&Magnesium or Wire-arc&Mg or Wire-arc&Magnesium, LPBF&Mg or LPBF&Magnesium or SLM&Mg or SLM&Magnesium, Web of Science (SCIE, SSCI, A&HCI, ISI Proceedings) databases (2024 version)).

The current magnesium AM techniques include selective laser melting (SLM, also known as laser powder bed fusion, LPBF), wire arc additive manufacturing (WAAM), jetting technologies (BJ), friction stir additive manufacturing (FSAM), and indirect additive manufacturing (I-AM) [27]. Among these techniques, WAAM and LPBF are more widely adopted. Over the past five years, the volume of publications on magnesium alloy produced by LPBF and WAAM has generally shown an increasing trend, indicating the potential and promising prospects for the applications of LPBF and WAAM (Figure 3). This paper will focus on the processes and influencing factors of each method.

## 2. Materials and Methods

### 2.1. Powder Bed Fusion (PBF) of Magnesium

**Figure 4:**
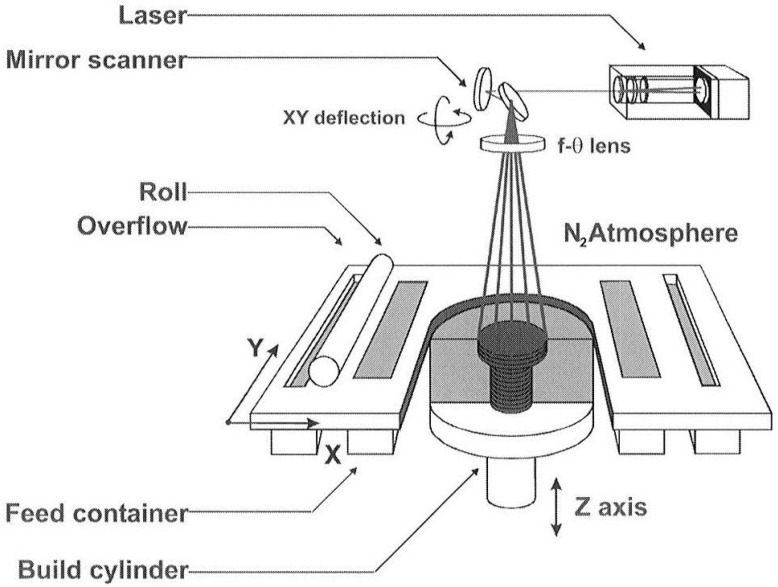
The schematic diagram of a PBF system [31].

Powder bed fusion is an AM process where thermal energy selectively fuses regions of a powder bed [28]. This bed consists of metal, polymer, or ceramic powders as the starting materials. An energy source is precisely guided over the powder bed to scan and melt targeted areas on the top layer. After melting the specified regions, the bed lowers to make room for a fresh layer of powder, which is then evenly spread over the previously fused layer. This repetitive process continues layer by layer until the intended structure is formed by building up multiple fused powder layers [25]. Figure 4 illustrates the schematic of the PBF process.

In magnesium alloy PBF, laser has been studied most deeply. The laser works by focusing high temperatures briefly on tiny parts of the powder bed, causing the powder to melt quickly. This short heat flow process leads to rapid heating and cooling of the molten powder, which causes its rapid solidification phenomenon. This rapid solidification process makes subtle adjustments to the grain structure, thereby increasing the bearing capacity of the material under large loads [25].

There are many significant parameters, including powder size, energy density, laser power, scanning speed, and laser thickness. A method to identify key factors in magnesium PBF involves design of experiments (DOE). The statistical method of DOE cuts down on both cost and time, achieved by decreasing the quantity of experiments performed [29]. Gangireddy et al. [30] analyzed the factors influencing AZ31(Mg-Al-Zn alloy) PBF using DOE, and the results showed that the optimal printing setting of magnesium alloy usually occurred at a lower energy density. At higher energy density, the porosity is greatly reduced. At lower energy density, the final component density is higher, and the evaporation of alloying elements in the melt pool is lower [30].

Equation (1) illustrates the determination of the energy density (Ev) of lasers imparted to the magnesium powder.

(1) $$Ev=\frac{P}{S\cdot V\cdot T}$$

P, S, T, and V represent laser power, hatch spacing, layer thickness, and scanning speed, respectively. The formula indicates that different combinations of laser powers and scanning speeds could result in the same energy density [29].

#### 2.1.1. Selective Laser Melting

The predominant methods for AM processing of Mg alloys using lasers include LPBF and direct laser deposition (DLD), with LPBF being the most commonly used technique for AM of Mg [23].

**Figure 5:**
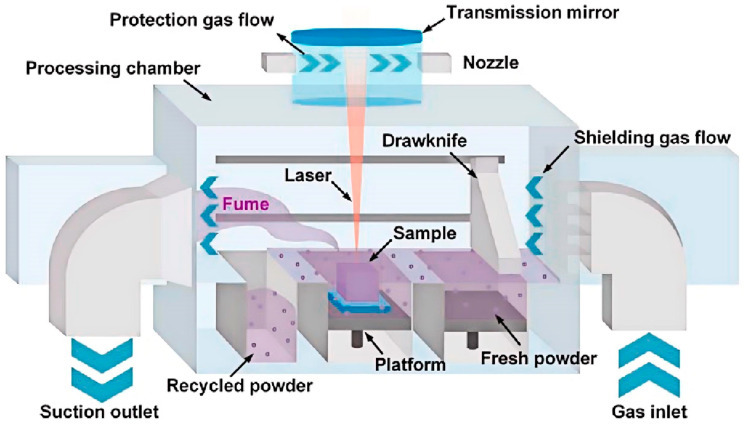
Schematic of LPBF process [33].

Figure 5 illustrates the schematic of LPBF. LPBF offers a convenient and versatile means for processing alloys and composite materials. There are two main routes for preparing composite powder in the LPBF process. The first involves melting a composite ingot and subsequently atomizing it to produce the powder. Alternatively, the matrix alloy is initially powdered and mixed with nano- or micron-sized reinforced particles to synthesize the composite powder. This latter approach offers significant advantages in practical production settings [1]. It is due to the substantial expense included in producing tailored atomized pre-alloyed powder, which is considerably higher than that for cast and wrought magnesium alloys [32].

Relative density (RD) is a crucial indicator for assessing the formation quality of LPBF components. Wei et al. [34] found that the content of zinc affects the solubility of magnesium alloy in LPBF. The study reveals that achieving high relative density, nearing 99%, is feasible solely when the Zn content falls below 1 wt.% or exceeds 12 wt.%. However, Mg-Zn specimens exhibited significantly lower corrosion resistance compared to the commonly utilized Mg-Y-RE alloy system in medical uses [35].

A well-defined grain morphology and minimal heat-affected regions are crucial for maintaining structural integrity. For composite materials, SLM technology’s rapid melting and solidification characteristics have several benefits. First, they significantly reduce the heat-affected zone, leading to improved precision in the production of zero parts. Second, these characteristics result in specimens with finer grains and higher mechanical strength. The cooling rate in the molten pool can reach 104–106 K/s, leading to a substantial refinement in magnesium’s microstructure. Furthermore, the formation of supersaturated solid solutions and metastable phases alters the material’s properties [36,37]. During the LPBF process, the presence of uniformly distributed Mesoporous Bioactive Glass (MBG) nanoparticles in the melting pool tends to hinder the movement of grain boundaries, thereby inhibiting crystal growth and promoting the formation of fine particles [38]. Additionally, LPBF’s rapid solidification characteristics effectively suppress grain growth. This rapid solidification helps mitigate the localized aggregation of reinforced particles in composite materials, caused by their large surface energy. It also facilitates uniform particle dispersion within the matrix, addressing issues of stress concentration and toughness loss that can lead to part failure. Moreover, due to the highest thermal gradient being in the direction perpendicular to the workpiece, the specimen’s microstructure exhibits anisotropy [23,39,40,41,42,43].

The inclusion of fine grains in Mg-based composites significantly enhances their mechanical properties. After using the intercept technique to measure grain dimensions, it was found that ZK60(Mg-Zn-Zr) alloy had an average grain size of 12.3 μm. In contrast, ZK60/BG and ZK60/MBG composites showed significantly finer grains, averaging 6.2 μm and 5.7 μm, respectively [38]. Bär et al. [44] reported that the average grain size of magnesium alloy produced by laser additive manufacturing is 4.7 μm, smaller than the grain sizes mentioned earlier. Regarding tensile properties, ZK60/BG and ZK60/MBG demonstrated significant improvements over ZK60, with ZK60/MBG achieving particularly high-yielding and ultimate tensile strengths. These enhancements can be attributed to the combined effects of nanoparticle reinforcement and grain refinement [45].

**Figure 6:**
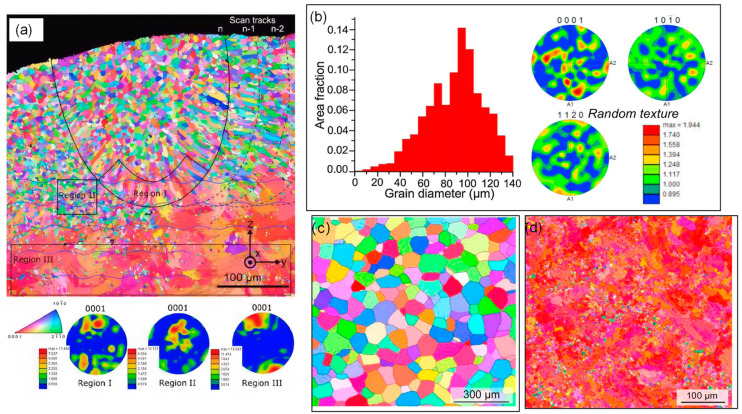
The grain orientation map of the LPBFed WE43 Mg alloy. (**a**) Fine, equiaxed, and randomly orientated grains in the last melt pool [44]; (**b**,**c**) figures revealing the grain structure and the random texture; (**d**) large, irregular-shape, and basal-orientated grains [47].

Zumdick et al. [42] observed refined equiaxed grains in the WE43(Mg-Al-Zn-RE alloy) Mg alloy treated by LPBF but noted abnormal grain growth caused by the temperature gradient in the deposited layer of the alloy. Conversely, Bar et al. [44] reported irregular and large basal grains in their study of LPBF-fabricated WE43. A key observation was that equiaxed grains with random texture were confined to the center of the final melt pool, surrounded by columnar grains and larger irregular grains (Figure 6). This transition from columnar to equiaxed grain structure during the continuous solidification process of the molten pool highlights the dynamic nature of grain formation in additive manufacturing [46].

There are several defects in the LPBF process, including improper powder melting, oxidation and evaporation, and residual stress. Specifically, magnesium poses unique challenges due to its low heat absorption, which causes it to reflect laser energy, leading to insufficient melting of powders [24]. Additionally, given the reactive nature and oxidation susceptibility of Mg powder, it must be stored in an oxygen-free environment and processed in an inert atmosphere [13].

**Figure 7:**
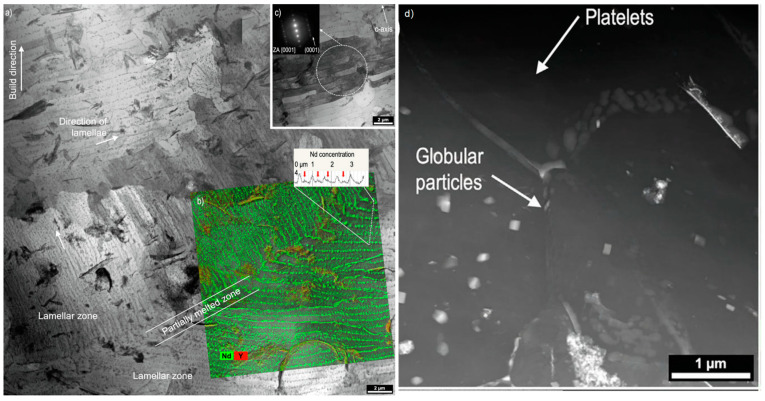
(**a**) The bright-field image of LPBF-processed WE43. The cross section shows a partially melted zone and a lamellar zone (labeled in the figure). The building direction of the specimen and the directions of the lamellae are indicated by white arrows. (**b**) A chemical mapping of the lamellar zone and partially melted zone reveals Nd-rich particles on the lamellae boundaries. The corresponding line scan shows varying Nd concentration across adjacent lamellae. Concentration peaks in the lamellae’s centers are indicated by red arrows. (**c**) BF-TEM image showing a similar crystallographic orientation of adjacent lamellae in the lamellar zone. (**d**) Heat-affected zone in LPBF processed WE43 STEM imaging [44].

A microstructural analysis of LPBF-fabricated WE43 revealed three distinct zones: partially melted, lamellar, and equiaxed (Figure 7) [44]. Heat exposure during the LPBF process alters the morphology of secondary phases, precipitates Nd-rich intermetallics, enhances grain growth, and creates a pronounced texture. One significant advantage of LPBF over traditional casting methods is the reduction in grain sizes, which can improve the material’s mechanical properties.

LPBF, with its high precision, high degree of design freedom, and excellent manufacturing capability for complex structures, heralds the prospect of a wide range of applications in the fields of aviation, aerospace, automotive, and consumer electronics. The technology is not only capable of manufacturing lightweight parts with complex internal and external geometries but also optimizes the microstructure and properties of materials through the fine tuning of the process parameters to achieve form-fit co-design. In particular, in the biomedical field, the biocompatibility and degradability of magnesium alloys combined with LPBF technology provide a new way to fabricate biological implants with complex structures. However, challenges such as evaporation of magnesium alloys at elevated temperatures and porosity control need to be overcome by optimizing process parameters and taking appropriate protective measures.

##### Magnesium Powder

Typically, the powder used in PBF ranges from 20 μm to 150 μm in size, with a tendency towards the lower end of this spectrum [29]. The elevated surface energy of magnesium powder due to its small particle size makes it prone to oxidation and presents challenges in depositing it in layers. To reduce sensitivity to oxidation, magnesium is often alloyed with other elements. This alloying not only reduces oxidation susceptibility but also influences the grain structure, heat resistance, and strength of the material [48]. As mentioned above, the size of powder particles affects both the degree of oxidation and the quality of depositions, with smaller particles being more reactive and potentially more challenging to handle [25,49].

Powder recycling can exacerbate oxidation issues in additive manufacturing processes. Preheating addresses this by reducing the heat flux between the powder and the heat source, resulting in smoother and flatter depositions. Additionally, preheating enhances the wettability of magnesium during 3D printing and improves surface roughness. These optimizations collectively enhance the overall quality and performance of the printed magnesium material [25,50].

##### Layer Thickness

The thickness of layers significantly impacts printing speed. Thin layers necessitate more frequent powder spreading over the bed, while thicker layers may lead to inadequate melting. Balancing layer thickness is crucial for optimal printing outcomes. Depositions of pure Mg powder were smooth only up to a layer thickness of 0.25 mm. Increasing the layer thickness can help reduce oxidation [25,50].

##### Degradation Behavior of LPBF Mg Alloys

To determine the corrosion current density of LPBF pure Mg in Hank’s solution, Niu et al. [51] investigated and found that its corrosion current density (icorr) ranged from 74 to 177 μA/cm^2^. It is significantly higher than the corrosion current density of 23.6 μA/cm^2^ obtained from the traditional as-cast specimens under the same conditions [52]. Corrosion resistance is compromised by loosely melted magnesium and sintered magnesium powder clusters. Similarly, rapid degradation phenomena have been observed in LPBF Mg alloys. Yin et al. [53] analyzed and found the degradation behavior of LPBF WE43 and cast WE43 in Hank’s solution, revealing that even with optimized process parameters, LPBF WE43 exhibited faster degradation. Due to repeated remelting during LPBF, secondary phase precipitates form in the microstructure, causing severe galvanic corrosion. According to Esmaily et al. [47], LPBF WE43 undergoes higher degradation in NaCl than cast WE43, resulting in structural integrity loss. Overall, LPBF processing can lead to degradation issues in Mg alloys due to microstructural changes caused by the process.

##### Energy Density and Vaporization

As the energy density increased, the relative density of the material decreased [16]. With pure Mg powder with spherical particles averaging 24 μm in size, depositions with a density of 97.5% were achieved at a low energy density of 155.56 J/mm^3^ [51]. WE43, a magnesium alloy primarily composed of yttrium and neodymium, exhibited a relative density of 99.4% when printed at a density of 238 J/mm^3^ [44]. A Mg-Al-Zn alloy (AZ61) with a mean particle size of 48 mm requires energy at 156 J/mm^3^ for creating structures with a relative density of 99.4% [54]. This demonstrates the potential of Mg-based alloys in additive manufacturing for achieving high-density depositions.

**Figure 8:**
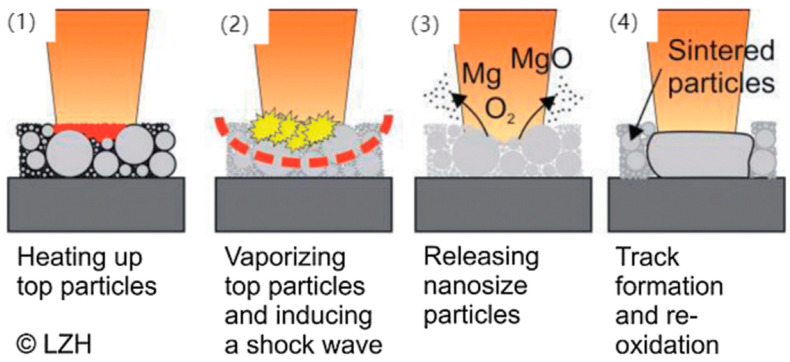
Evaporation products formed during SLM processing of magnesium alloys. (1)–(4) describe the machining process of SLM [60].

Mg exhibits a relatively low evaporation temperature of 1091 °C compared to other metals. Under pressure, molten material splatters outward in the melt pool, resulting in a low-density structure (Figure 8). Consequently, magnesium alloys experience chemical compositional changes and inevitable porosity during the LPBF process [44,55]. Wei et al. [56] found that the evaporation rate of Mg in AZ91(Mg-Al-Zn alloy) alloy increases significantly with Ev. An optimal Ev for AZ91 is approximately ~60 J/mm^3^; higher values result in excessive Mg evaporation, whereas lower values cause a smaller melt pool size and accelerated Mg evaporation due to localized high temperatures and poor powder thermal conductivity. Magnesium alloy ZK60, containing Zn and Zr, undergoes significant vaporization of magnesium and zinc elements when exposed to 1250 J/mm^3^ of energy [16]. The LPBF processing window for Mg alloys is restricted to minimize compositional changes and prevent severe evaporation. Although minor defects may be tolerable, it is crucial to avoid significant issues like hot tears and cracks during LPBF [57].

The energy density range in additive manufacturing is significantly influenced by factors such as layer thickness and powder quality. Adjusting process parameters for interior and surface layers is crucial to achieve dense depositions with superior surface quality. Furthermore, incorporating bioactive glass in PBF printing of Mg-Zn-Zr alloy (ZK30) enhances corrosion resistance in vitro [54,58]. Studies have shown that optimal printing conditions for the Mg-Al-Zn alloy AZ91D involve a 200 W power setting and a scanning speed of 0.09 m/min, leading to an energy density range of 83 J/mm^3^ to 167 J/mm^3^. Investigations indicate that the smoothest depositions occur at an energy density of 122 J/mm^3^ [59]. 

### 2.2. Wire Arc Additive Manufacturing

**Figure 9:**
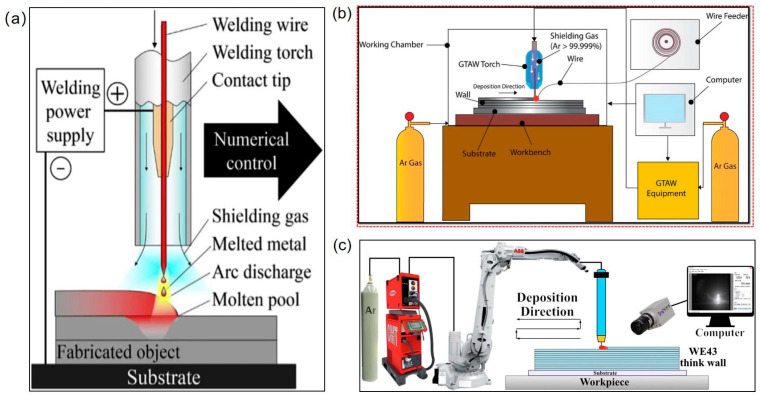
(**a**) The material deposition for wire arc additive manufacturing [27], (**b**) the symmetric representation of the GTAW–WAAM process [65], (**c**) CMT–WAAM system [12].

Employing the direct energy deposition (DED) method, wire arc additive manufacturing systems function by layer-by-layer cladding using wire as the raw material and an electric arc as the heat source. A constant feed of metal wire is melted by the arc and deposited onto the previously laid layers. WAAM employs three wire-based welding techniques: tungsten inert gas (TIG), plasma arc welding (PAW), and metal inert gas (MIG) [61,62,63]. Figure 9a illustrates the schematic of WAAM. WAAM can be categorized into cold metal transfer (CMT or gas-shielded metal arc welding (GMAW)) and gas tungsten arc welding (GTAW) depending on the necessity of a non-consumable tungsten electrode in the system (Figure 9b,c) [64].

Compared to LPBF, WAAM offers benefits such as high deposition rates, environmental friendliness, efficient material utilization, and low equipment costs [23,27]. Consequently, WAAM has emerged as a promising approach for manufacturing near-final shapes on a large scale [66,67,68,69,70]. Moreover, the handling of magnesium powders is mitigated in WAAM processes to reduce potential safety risks, using Mg wire as raw material instead of Mg powder. Working inside a chamber is unnecessary due to the welding gun’s gas shielding. For example, in GTAW, a non-consumable tungsten electrode generates an arc between the electrode and the workpiece within an inert gas environment, typically helium or argon [71,72]. This inert atmosphere effectively shields the material from corrosion and oxidation [68]. Additionally, the production of Mg alloy filaments involves wire drawing rather than atomization, leading to significant cost reductions in feedstock materials, including production, storage, transportation, and powder management [23]. WAAM also achieves high manufacturing speed by melting wire as filler metal, using commercial arc welding systems and wires, thereby eliminating the need for additive manufacturing-specific facilities and materials. Furthermore, it produces products without plastic deformation, requiring only minimal machining of near-net products [73].

Due to their excessive heat, which causes warping and melt-back issues due to magnesium’s low melting and evaporation points, MIG and TIG welding are unsuitable for WAAM of Mg alloy. CMT is currently the most suitable technique for Mg alloy WAAM due to its lower heat output. For forming magnesium alloy structural parts, this method effectively mitigates issues like thermal stress, thermal cracking, spattering, and grain growth in the Mg alloy WAAM process owing to its characteristic of low heat input [23,74]. However, energy and mass transfer are intricately linked in the CMT process. Even with identical transition metals, the local thermal processes vary significantly under different operational modes, potentially leading to diverse microstructures and mechanical properties [75].

**Figure 10:**
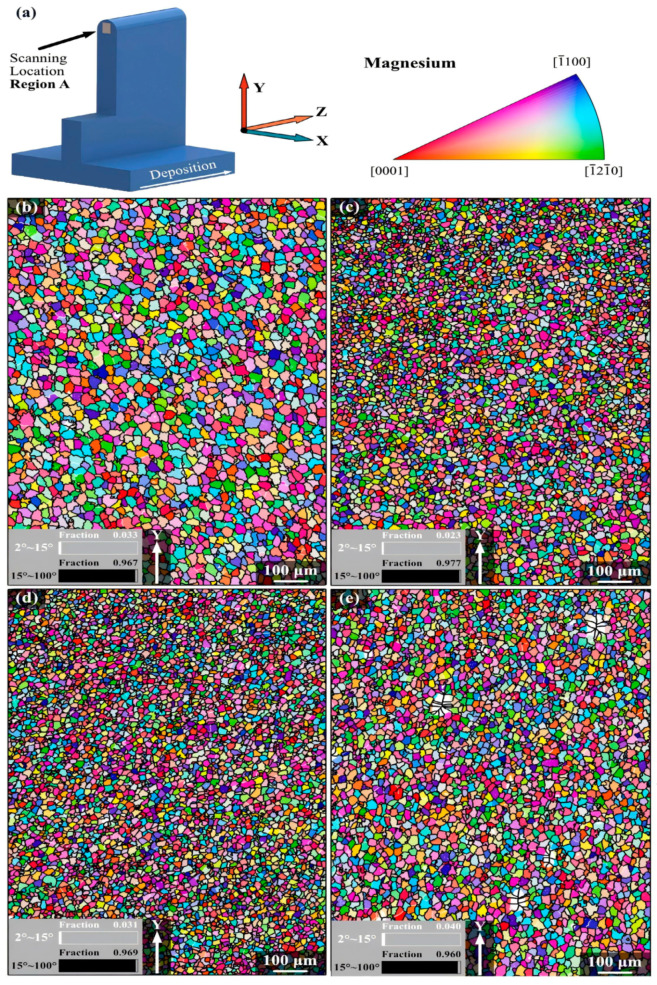
The EBSD inverse pole figure orientation maps, grain size distribution figures, and pole figures of WE43-Mg cross-sectional specimens deposited by different CMT process modes in the top region: (**a**) Scanning location region A; inverse pole figures with reconstructed grain boundaries of (**b**) CMT, (**c**) CMT-P, (**d**) CMT-ADV, (**e**) CMT-PADV [12].

The top region of all four specimens exhibited predominantly equiaxed grains, as illustrated in Figure 10b–e. All four CMT process modes (CMT, CMT-Pulse + Advance, CMT-Advance, and CMT-Pulse) successfully produced multi-layer WE43-Mg single-pass components. In the top region, the deposited components showcased equiaxed grains of consistent size, while the middle region exhibited a layered structure. CMT–WAAM’s remelting effect refined the grains, leading to fine-grained areas between layers. The width of the remelted fine grain zone decreases as the line energy increases. Among the modes, the CMT pulse mode demonstrated superior results attributed to its ability to mitigate the common defect of low porosity in the as-deposited WE43-Mg. CMT-PADV showed higher porosity compared to the other modes [12].

Path simulation software, the wire feed system, communication lines, execution actuator, and welding power constitute essential components of a WAAM system. Metal parts are fabricated by precisely controlling the actuator’s displacement, speed, and position, replicating the molten pool through point-by-point control. Factors such as actuator accuracy, welding parameters, mode, and arc heat stability significantly influence part accuracy. The wire feed rate corresponds to electrode consumption, while energy input is determined by voltage and current. The deposition rate (DR) of WAAM is mathematically described by a specific equation (Equation (2)), as detailed in the referenced paper [23].

(2) $$DR=60\cdot \rho \cdot {\bar{v}}_{WF}\cdot A_{ww}$$

where ${\bar{v}}_{WF}$ is the mean wire feed speed, $A_{ww}$ is the weld wire cross-sectional area, and ρ represents the density of the feedstock alloy (1.73 g/${cm}^{3}$ for Mg) [76].

**Figure 11:**
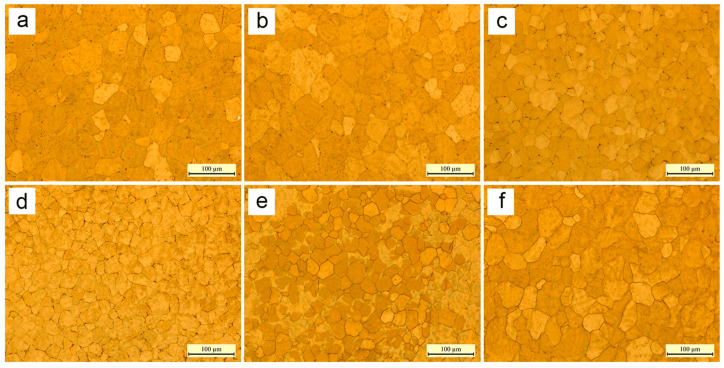
Microstructures of AZ31 deposited by different pulse frequencies: (**a**) 500 Hz, (**b**) 100 Hz, (**c**) 10 Hz, (**d**) 5 Hz, (**e**) 2 Hz, and (**f**) 1 Hz [75].

The mechanical properties such as strength, ductility, hardness, corrosion resistance, fatigue endurance, and creep resistance are significantly influenced by the grain size. According to Hall–Petch’s equation, larger grain size inversely affects the tensile strength of Mg alloys [64,77]. Razavi et al. [78] observed a decrease in the grain size of the AZ31 alloy with increasing pulse frequency, followed by a slight increase. The WAAM process of the AZ31 magnesium alloy exhibited the smallest grains and highest tensile strength at arcing frequencies of 5–10 Hz. Lower or higher frequencies resulted in coarser grains. Increasing deposition speed and feed led to smaller grains due to improved nucleation homogeneity and grain refinement from rapid bead cooling in the WAAM process. This rapid cooling was achieved by precise control of the heat input and localized melting of the metal wire induced by the arc generated by a short circuit [24]. Simultaneously, smaller grains were observed as the deposition rate and feed increased. The quality of depositions was significantly affected by arc frequency during the TIG WAAM process. Grain sizes became finer within the 5–10 Hz frequency range due to the higher deposition rates. Outside this range, an increase in grain size was observed (Figure 11) [25,29,75]. However, due to inadequate molten pool control, the average grain size of the WAAMed AZ31 alloy was 21 µm under optimal conditions [75].

Extensive research has been conducted on AZ-series Mg alloys(Mg-Al-Zn alloy) produced via wire-arc DED to address defects, refine microstructure, and enhance performance. While the ductility of these alloys has reached standards comparable to wrought states, their strength remains insufficient and requires further enhancement. This deficiency primarily arises from the low quantity of nano-sized Mg_17_Al_12_ precipitates on the basal planes [66,79,80,81,82,83,84,85].

**Figure 12:**
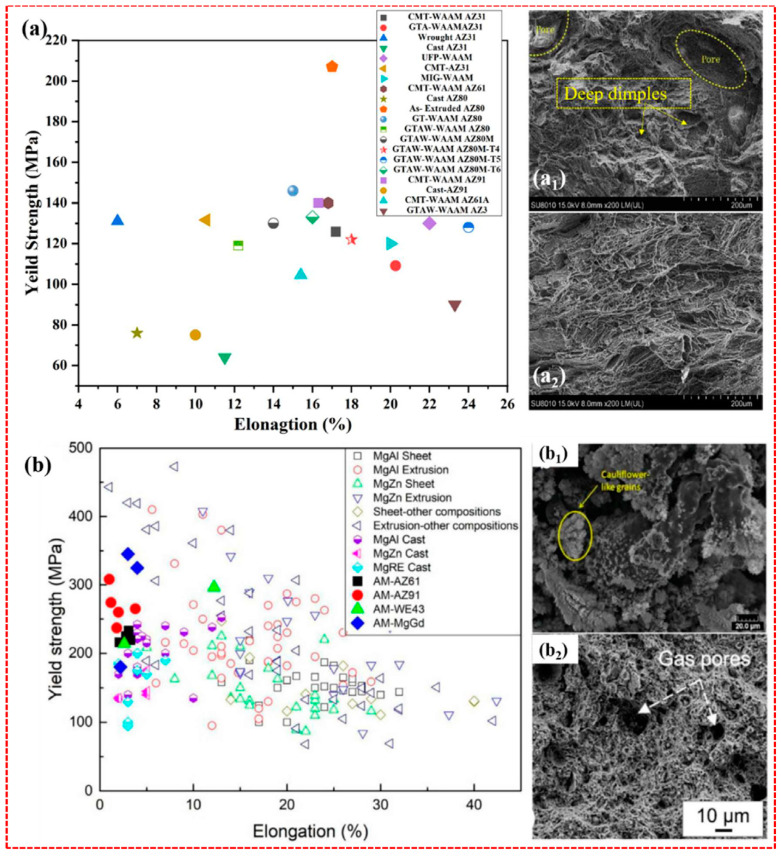
Comparison of mechanical properties between (**a**,**a_1_**,**a_2_**) WAAMed Mg alloy and (**b**,**b_1_**,**b_2_**) LPBFed Mg alloy [24].

Figure 12 shows the comparison of mechanical properties between WAAMed Mg alloy and LPBFed Mg alloy. Remarkably, WE43 and GW63K(Mg-Gd-Y alloy) alloys demonstrated exceptional strength exceeding 300 MPa, albeit with a limited elongation ranging from 5–7%. The restricted ductility in Mg-RE alloys is due to dense nanoprecipitates that impede dislocation slip [86]. In contrast, Mg-based products manufactured using the WAAM technique display significantly higher ductility than powder-based AM methods. For example, WAAM-AZ91 exhibits an elongation of 16%, while SLM-AZ91 demonstrates less than 2% elongation [87]. Research by Tong et al. [88] has revealed that the porosity and rare earth oxide content in WE43 alloy processed through wire-arc DED is significantly lower compared to LPBF, resulting in enhanced ductility of 10.2%. Similarly, Wei et al. [89] achieved a ductility of 9.9% in wire-arc DED-processed Mg-3.2Y-1.6Nd-0.5Zr alloy. These results indicate that wire-arc DED is an effective method for producing Mg-RE-Zr alloys with improved ductility. In their investigation, a Mg-3.2Gd-0.6Y-0.5Zr alloy, with a similar alloying composition to commercial AZ31, was processed using wire-arc DED additive manufacturing coupled with TIG welding. Tensile testing revealed that this alloy exhibited high ductility, surpassing most reported additive-manufactured and wrought Mg alloys. This demonstrates the potential of the wire-arc DED process in fabricating Mg alloys with superior mechanical properties. The enhanced ductility of the DED specimen in Mg-RE-Zr alloys can be attributed to several factors. First, the inclusion of Gd/Y elements and the fine grain structure played a significant role in improving ductility by enhancing the activity of non-basal slip. Second, the fine grain structure suppressed twinning and promoted coordinated deformation among grains. Finally, the ductility was further enhanced by reducing microscopic damage through refining second-phase particles. These combined effects led to the exceptional ductility observed in the DED specimen [90].

The exceptional ductility and impact toughness of Mg-RE-Zr alloys originate from their significant plastic deformation capability and fine equiaxed-grain structure. A study about the correlation between microstructure and dislocation slip/twinning behaviors aimed to elucidate these characteristics. Owing to the near-rapid solidification conditions and the high molten pool superheat leading to Zr particle dissolution, the DED process induced multiple grain refinement effects, ultimately resulting in a fine grain structure with an average size of 12.3 ± 7.4 μm, considerably finer than that of the as-cast specimen [90]. Moreover, a new Mg-4.4Gd-2.2Y-1.0Zn-0.5Zr alloy (yield strength of 157 ± 1.15 MPa, ultimate tensile strength of 288 ± 2.52 MPa, and elongation of 17.1 ± 0.32%) exhibits exceptional strength–ductility synergy achieved through heat treatment-induced precipitate manipulation. By contrast, Mg-Gd series alloys produced via L-DED exhibit strengths exceeding 250 MPa but with limited elongation. Wire-arc DED offers a rapid and efficient method for manufacturing large-sized components with intricate geometries and distinctive microstructures [79,80,91].

In comparing the WAAM and LPBF additive manufacturing processes, the heat source nature and distribution resulted in distinct microstructures, leading to variations in grain size and cooling rates. In the WAAM process, heat is generated by an arc from an electrical short circuit, creating an asymmetric heat source distribution describable by a double ellipsoidal model [92]. Conversely, in LPBF, heat is generated by a solid-state Nd-YAG laser, resulting in a symmetric heat distribution described by a Gaussian model [93]. These processes yield significantly different microstructures due to their distinct heat sources and resulting cooling rates. For instance, LPBF-processed Mg alloys display finer grains owing to a high cooling rate of about 40 K/ms [94]. However, in thin-walled structures, the grain size typically increases from the bottom to the top section [95]. This phenomenon arises from heat accumulation from preheating the previous layer, reducing the thermal gradient and resulting in larger grain sizes in the deposition direction [24,53]. Yang et al. [66] studied the microstructural evolution of the thin-walled longitudinal section of WAAMed AZ31 alloy and found that apart from the topmost layer, each layer exhibited a microstructure of vertical and steering columnar dendrites.

Compared to LPBF, WAAM-Mg has several significant drawbacks. First, the scaffold structures produced can be too coarse for medical applications, limiting their use. Second, the supply of suitable wires is limited due to the lack of customized alloy options. Finally, the procedure is highly complex, as magnesium can only be welded using AC techniques, presenting more challenges than initially expected [23]. Additionally, the WAAM process has particular disadvantages when manufacturing Mg-Al alloys. The CMT heat source, because of its large molten pool, tends to generate columnar grains, which may compromise properties [66,96]. However, this can also be advantageous. Wu et al. [97] employed the WAAM method alongside the CMT technique, leveraging the columnar dendritic structure, to produce a thin-walled AZ31 alloy component. This approach took advantage of the unique characteristics of CMT for efficient and precise alloy production. Element segregation is inevitable during the complex solidification process [98]. Under multiple thermal cycles, Mg-Al alloys are susceptible to hot cracking and liquation cracking.

Various auxiliary processes, such as cryogenic cooling [79], interlayer ultrasonic impact [99], arc oscillation [100], and laser shock peening [80], have been explored to enhance the mechanical properties of Mg-Al alloy components. These processes have significantly improved grain refinement, stress regulation, and porosity inhibition. However, they may limit component size, extend manufacturing time, or necessitate changes to component shape. Therefore, it is essential to conduct further exploration of auxiliary processes and determine the most suitable manufacturing strategy for Mg-Al alloys [101].

In summary, WAAM has shown great potential in aerospace, marine, and architectural fields due to its low cost, high deposition efficiency, and suitability for the manufacturing of large-size complex structures. The technology is capable of achieving near-net-shaping and significantly improving material utilization. However, defects such as thermal cracks and porosity that may occur during the WAAM process as well as the relatively low forming accuracy are key issues that need to be addressed for future development. The forming quality and accuracy of WAAM technology can be effectively improved by optimizing the process parameters, selecting suitable welding wires, and conducting subsequent processing treatments.

### 2.3. Friction Stir Additive Manufacturing

Friction stir additive manufacturing is a sheet lamination process where layers of material are fused through frictional heat generated by a rotating tool, inducing plastic deformation and fusion. While this technique offers high strength and ductility, porosity remains a significant challenge [102]. At elevated tool speeds, the increased frictional heat forces material outwards, introducing residual stress due to thermal gradients [25]. Solid-state additive manufacturing methods also utilize friction stir processes for plastic deformation without melting. The earliest application of FSAM for Mg alloys was reported in 2014. Since then, several Mg alloys have been developed using FSAM or similar friction-based methods [102,103,104,105,106].

**Figure 13:**
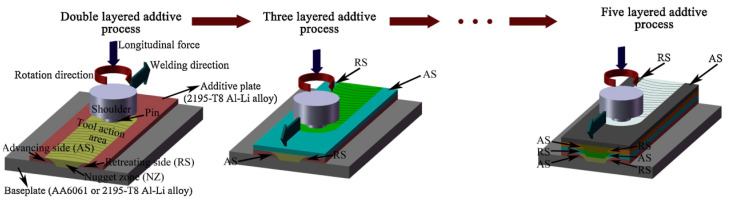
Schematic illustration of FSAM process [108].

Figure 13 illustrates the schematic of FSAM. FSAM distinguishes itself by keeping the alloy solid throughout the welding process. The process involves inserting a non-consumable rotating tool with a specially designed pin and shoulder into the overlapping sheets or plates intended for joining. Subsequently, the tool moves along the joint line, generating the necessary welding heat through frictional contact between the shoulder and the workpiece, accompanied by significant plastic deformation induced by the motion of the pin. The material moves from the front to the rear of the pin, facilitating the formation of a weld nugget whose macro shape is determined by the pin’s geometry. As a result, joints are formed without melting, ensuring a solid-state welding process [102,107].

At present, friction stir-based additive manufacturing of Mg alloys is conducted under atmospheric conditions, leading to minor oxidation, especially at layer interfaces. Implementing the process in an inert atmosphere could mitigate this oxidation concern. Zeng’s research compares the grain sizes of Mg alloys produced through friction stir-based AM with those of Mg alloys primarily used as plates or feed materials. The results show significant grain size refinement and a more equiaxed, uniform grain distribution in the deposited findings, even when utilizing a coarser-grained feedstock. Significantly, the grain sizes achieved through FSAM and another AFSD are comparable to those obtained through LPBF of Mg alloys. This suggests that friction stir-based additive manufacturing techniques can effectively produce Mg alloys with refined grain structures, potentially improving their mechanical properties [23].

Utilizing FSAM, adapted from friction stir welding (FSW), Palanivel et al. [102] successfully fabricated the high-performance WE43 magnesium alloy. The process notably enhanced the alloys’ mechanical properties after aging, attributed to the solid solution of solute atoms within the α-Mg matrix and the precipitation of strengthening phase particles [109]. However, hook defects may occur at the overlapping interfaces of multi-layer plates during the FSAM process [108]. Friction stir processes exhibit a microstructure consisting of three distinct regions, the stir zone, thermomechanically affected zone, and heat-affected zone, due to temperature gradients and spatial strain distribution. Operational parameters such as tool speed, traverse speed, plunge depth, tool geometry, and feed rate define the processing windows. Achieving optimal heat input is crucial to avoid defects, with friction stir processes typically operating at temperatures ranging from 0.6 to 0.9 of the materials’ melting point. Maintaining operating temperatures between 0.8 and 0.9 of the solidus temperature of Mg alloys can aid in producing defect-free structures [23,105,106,110,111].

**Figure 14:**
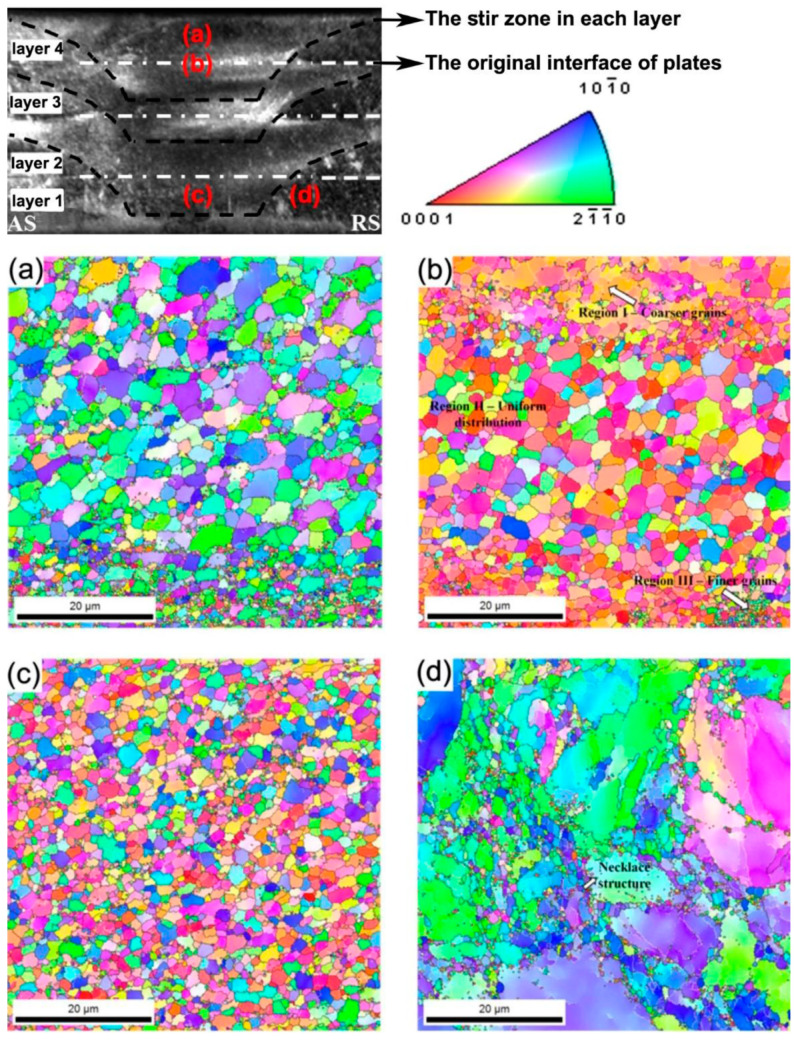
The EBSD orientation map of the WE43 alloy built by joining four layers of 1.7 mm thick sheets via the FSAM method, showing the distribution of grains in (**a**) the top layer (layer 4), (**b**) the sandwiched microstructure at the interface of layer 3 and 4, (**c**) the bottom layer (layer 1), and (**d**) a representative thermomechanically affected zone. Adapted from [102].

The EBSD inverse pole figure maps illustrated in this study depict the variations in grain size across four layers of WE43 alloy produced via friction stir additive manufacturing. Fine equiaxed grains, resulting from dynamic recrystallization, were evident within the stir zone (Figure 14a,c). Each layer deposition created a thermomechanically affected zone and a heat-affected zone in the underlying layer. Notably, the interface between these layers exhibited a sandwiched microstructure characterized by coarser grains at the top, finer grains with a more uniform distribution in the middle, and very fine submicron-sized grains at the bottom (Figure 14b). This microstructural transformation, influenced by the spatial distribution of deformation and temperature along the build direction, remains partially understood but significantly impacted corrosion resistance. Moreover, the thermomechanically affected zone exhibited a necklace-type structure, indicating recrystallization at grain boundaries (Figure 14d). Grain size variations across the layers have also been observed in WE43 alloy fabricated using the alternative friction stir-based method, AFSD [103]. The presence of such microstructural heterogeneity may lead to material property inhomogeneity, potentially compromising the strength and fatigue performance of friction stir-based additively manufactured Mg alloys [23]. Understanding and controlling these microstructural variations is crucial for optimizing the performance of these alloys in additive manufacturing processes.

However, FSAM also exhibits several disadvantages. First, the design flexibility of FSAM is constrained by several factors, including the machine’s dimensions and tool traverse speed, which have an impact on production scale and build speed. This limitation significantly hinders the ability to scale up production and increase fabrication speed. Second, clamping the material poses significant challenges. Third, tool wear progressively occurs over time. Fourth, significant residual stresses may accumulate within the build. In addition, insufficient material flow under the friction stir tool can lead to varying microstructures between the top and bottom layers. This issue encompasses defects from previous lap joints, the vertical overlap of weld nuggets, cracks along the stir zone, and the interface between the Thermo-Mechanically Affected Zone (TMAZ) at the retreating side bottom [23,102].

In summary, FSAM offers a new solution for the additive manufacturing of magnesium alloys with its high-performance manufacturability and defect reduction. FSAM technology is based on a stirring friction dynamic recrystallization process, which is capable of obtaining an ultra-fine grain organization and improving the performance of the part. At the same time, the technology is suitable for the manufacturing of larger volume components, which has potential applications in aerospace and automotive fields. However, the stability of the FSAM process and the control of defects, such as porosity and banding, still need to be further studied and optimized.

### 2.4. Binder Jetting

Binder jetting AM, also known as “inkjet 3D printing,” utilizes dispersed particles on a powder bed to systematically construct the desired structure layer by layer. This process involves the meticulous deposition of droplets of a liquid binder. Within the realm of binder jetting techniques, both continuous inkjet (CIJ) printing and drop-on-demand (DOD) methods are utilized [112,113]. One notable advantage of binder jetting is its ability to manufacture structures at ambient temperature, facilitating the incorporation of organic, biologically active, or hydrated molecules [114]. Ideally, the particle size ranges from 15 to 35 μm, and the approximate resolution is 20 to 30 μm [25,115,116].

**Figure 15:**
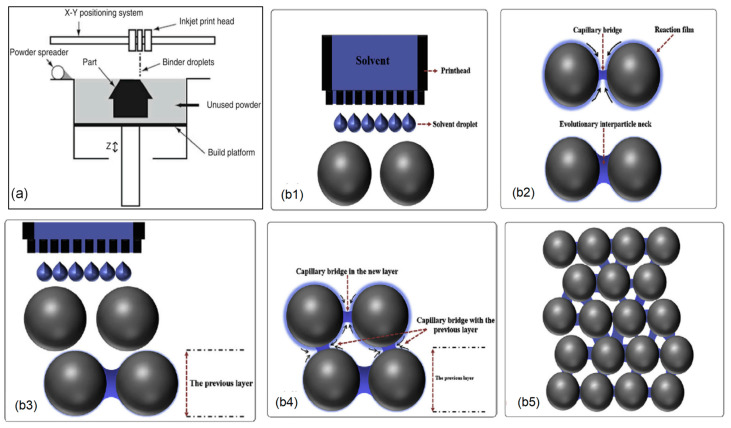
(**a**) Process flow diagram for binder jetting printing [123]; (**b**) Principle of binder-less jetting: (**b1**) solvent deposition, (**b2**) development of capillary bridges among wet particles, (**b3**) pre-addition of next powder layer, (**b4**) capillary action forming bridges between particles in new and previous layers, and (**b5**) fully developed solid structure formed after drying and sintering [29].

Figure 15 illustrates the schematic and fundamental principles of binder jetting. The process starts by 3D printing green parts in the desired shapes, which are then transformed into functional, densified components [117]. During the initial stage, powder particles in each subsequent layer are bonded through adhesion or chemical reactions, facilitated by polymeric binders or targeted solution deposition. Throughout this process, the printed object is consistently supported by the powder bed to achieve the required shape and structure, all at room temperature, thus preventing rapid oxidation even in an air atmosphere. Subsequently, the binder is removed through debinding, and the printed green parts undergo sintering in a protective atmosphere to prevent rapid oxidation. This step also involves densification processes, which may include sintering [118,119] or hot isostatic pressing [120]. These subsequent processes enhance the mechanical properties and longevity of the final part, making it suitable for a wide range of applications [117,121,122].

The primary challenge in binder jetting AM of Mg alloy is achieving sufficient densification. The densification of magnesium alloy parts produced by binder jetting technology heavily relies on material migration and diffusion. This requirement arises due to the propensity of magnesium powders to develop a MgO film on their surfaces. In contrast to Fe and Cu, the diffusivity rate of magnesium oxide is significantly lower (~1.2 × $10^{-42}$ m^2^s^−1^ at 189 °C) compared to its self-diffusivity rate. In sinter-based additive manufacturing of magnesium alloys, the initially printed part consists of a loosely packed arrangement of primary powder particles. The native oxide film either remains intact or thickens due to interactions between the binder materials and magnesium powder. Consequently, the magnesium oxide film poses a substantial hindrance to mass transport and the formation of sintering necks between magnesium alloy powders during the solid sintering process [124,125,126,127,128].

Due to the use of binding liquid in the BJ method, meticulous attention must be paid to the compatibility of binders with powders and their adhesive properties. Additionally, various critical factors, including print orientation, print speed, scanning technique, heater power ratio, and powder distribution, significantly influence BJ or inkjet 3D printing. Given their profound impact on printing efficiency, aspects related to powder spreading, such as roller speed and traverse speed, are also crucial. Therefore, a comprehensive understanding and careful management of these variables are essential for the success of BJ processes [129,130].

According to Equation (3), the theoretical binder saturation, S (in%), may be calculated.

(3) $$S=\frac{1000\cdot V}{(1-(\frac{PR}{100}))x\cdot y\cdot z}$$

V and PR denote the binder volume per drop measured in picoliters (pL) and the packing rate expressed as a percentage (%), respectively. Additionally, x, y, and z represent the distance between binder droplets in millimeters (mm), layer thickness in mm, and other pertinent parameters. By optimizing factors such as the degree of binder saturation, one can attain the desired mechanical characteristics and surface quality of the final printed structure [130].

The binder plays a pivotal role in binder jetting technology, exerting significant influence over the entire build cycle and subsequent processes, including curing, powder removal, debinding, and sintering of the printed component. Its physical attributes, surface tension, viscosity, and material compatibility, are critical for ensuring precise and stable printing outcomes. An optimized binder formulation yields uniform droplets capable of effectively penetrating the fine pores of the powder bed, thus facilitating the creation of robust and uniform bonding necks. This ensures the printed part possesses sufficient strength post-curing, thereby mitigating the risk of damage during its removal and transfer processes. Conversely, inappropriate binders may yield irregular and unstable droplets that struggle to permeate the powder effectively, resulting in weak initial bonds that are difficult to remove. Moreover, such a scenario could potentially lead to nozzle clogging or even damage to the inkjet printhead [128]. The commonly used binder is an aqueous organic binder. Su et al. [117] employed a binder of deionized water and various low molecular weight alcohols that volatilize completely at 350 °C. Li et al. [128] used an internally developed aqueous binder, primarily composed of glycerol and water. This binder effectively facilitated the creation of a homogeneous and stable bonding neck with magnesium alloy powder, ensuring robust construction. Moreover, it streamlined the essential processes of depowdering, debinding, and sintering the green specimens, facilitating a seamless transition from the printing stage to the final product.

The sintering process plays a pivotal role in enhancing the density and mechanical properties of Mg alloy specimens. Initially, low-temperature sintering in air consolidates individual MgO films, establishing a net-like framework structure. This structure preserves the specimen’s shape during subsequent high-temperature sintering under an Ar atmosphere, where the sintering temperature exceeds the liquidus temperature. During this phase, alloy powders transition into a high-viscosity liquid, effectively interconnecting and filling pores. As a result, the strength of the specimens significantly improves, approaching that of as-cast Mg alloy [117]. The sintering process includes intricate phenomena: particle rearrangement [131], pore elimination [132], and grain growth [133]. Several factors influence magnesium alloy sintering: initial bulk density, heating rate, sintering temperature, and holding time [134,135]. Improper sintering temperature selection can result in inadequate part density and mechanical properties [136,137]. An optimal sintering temperature facilitates viscous flow and material migration, promoting densification. Conversely, an inappropriate sintering temperature may impede densification efforts. Generally, higher sintering temperatures correspond to increased specimen density post-sintering. Conversely, insufficient particle diffusion due to low temperatures can hinder high-density part formation [138,139]. Conversely, excessively high sintering temperatures can increase the liquid phase and reduce viscosity, resulting in specimen swelling and distortion [140,141]. Gupta asserts that during single-step sintering, it is crucial to maintain the sintering temperature below the point where the liquid phase content reaches 24% volume, as exceeding this threshold may lead to swelling phenomena [132,133,138,140]. Simultaneously, the sintering temperature also impacts porosity. Initially, as the sintering temperature increases, the pores in the specimen decrease, followed by an increase [117].

Sintering aid can improve the density and physical properties. The study unveiled that the incorporation of sintering aid into Mg powder substantially increased its densification rate by up to 25%. Compared to the monolithic Mg counterpart, the Mg-0.2 wt% alloy showed a relative density enhancement of approximately 7%. Furthermore, tensile strength increased by nearly 30%, compressive strength improved by approximately 15%, the elastic modulus rose by about 18%, and elongation surged by an impressive 185%. These improvements are due to the interaction mechanisms between calcium and the Mg powder, its MgO film, and other alloying elements. Previously, the Ca-assisted sintering technique was employed in the metal injection molding of Mg-Ca blends [142,143]. However, this sintering method faces significant challenges, primarily related to the restricted options for chemical composition and achieving microstructural homogeneity [144]. Additionally, the sintering process significantly boosts the density and strength of the parts by employing a high-viscosity liquid phase to fill the pores. Su et al. [145] observed that the AZ91D Mg alloy, with 5 wt% Fe content, demonstrates exceptional ultimate compressive strength, reaching 440 MPa, nearly equivalent to the as-cast alloy. Furthermore, it exhibits a rapid degradation rate of 9091 mm/year, comparable to soluble Mg alloys containing rare earth elements but at a substantially reduced cost.

Salehi et al. [124,146] effectively utilized super-solidus liquid phase sintering to disrupt the oxide film in ZK60 Mg powder, facilitating faster mass transfer among particles. Subsequently, Dong et al. [147] applied this sintering technique in extrusion-based printed Mg scaffolds. Moreover, Li et al. employed a full liquid sintering approach to achieve densification by breaking the oxide film at high temperatures, enabling particle diffusion and migration. Their investigation unveiled that the density and mechanical properties of the Mg-9.08%Al-0.65%Zn alloy demonstrate a biphasic trend with increasing temperature, initially rising and then declining. Optimal properties were attained at 620 °C, exhibiting a density of 93.16%, compressive strength of 181.92 MPa, and tensile strength of 91.20 MPa. Additionally, the grain size increased by 18.56%, from 38.03 μm at 600 °C to 45.09 μm at 640 °C [128]. However, maintaining shape fidelity during liquid phase sintering poses significant challenges, particularly for geometrically complex and large parts, due to strict limitations on the maximum allowable liquid fraction [148]. The sintering kinetics of the as-printed Mg powder are directly influenced by the liquid content as well [146]. Salehi et al. [124,149] point out that these two considerations lead to a trade-off challenge between densification and shape fidelity.

Regrettably, there exists a paucity of research concerning grain growth and phase transformation throughout sintering. Present sintering techniques are plagued by extended cycles, subpar part density, and diminished mechanical properties. Consequently, it is imperative to investigate innovative sintering methodologies and gain a thorough understanding of the impact of sintering temperature on the properties of green parts to bolster the mechanical performance of magnesium alloys [128].

In summary, BJ technology has shown impressive potential and development prospects in the field of magnesium alloy additive manufacturing. With its high molding speed, the technology can rapidly build complex and fine three-dimensional structures, greatly shortening the product development cycle. At the same time, BJ has good adaptability to materials and can be flexibly applied to a variety of metal materials, such as magnesium alloy, which provides the possibility of diversified production of magnesium alloy products. In addition, the technology gives designers great freedom of design and can create complex shapes and internal structures that are difficult to achieve with traditional processes, further promoting the innovative application of magnesium alloys in aerospace, automotive manufacturing, and biomedical fields. Although the challenges posed by the chemical properties of magnesium alloys, such as their susceptibility to oxidation and evaporation, still need to be overcome in practical applications, BJ technology is expected to become an important force in the field of magnesium alloy additive manufacturing in the future through the continuous optimization of process parameters and the enhancement of material protection and to promote the continuous innovation and development of magnesium alloy applications.

### 2.5. Indirect Additive Manufacturing

Infiltration, categorized as an AM technique, is specifically tailored for producing Mg scaffolds. Its classification as an indirect AM method stems from its ability to fabricate final components or models by initially creating master prototypes, equipment, or other intermediates using non-AM techniques. In I-AM, a fusion of traditional and advanced tissue engineering methods is employed to design both the macro- and micro-characteristics of the final product [54,150,151,152,153,154].

**Figure 16:**
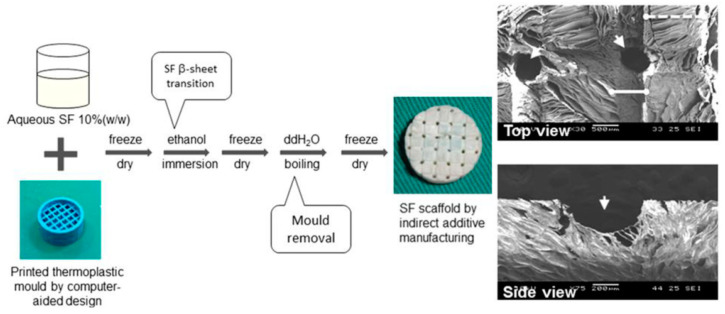
A printed thermoplastic mold through a computer-aided design was first manufactured and a silk fibroin (SF) scaffold was obtained using the indirect additive manufacturing technique. The top view (bar = 500 µm) and side view (bar = 200 µm) of the circled area in the SF scaffold by scanning electron microscopy are shown. Arrowheads indicate penetrating channels. Solid and dotted lines demonstrate channel and inter-channel regions, respectively [62].

**Figure 17:**
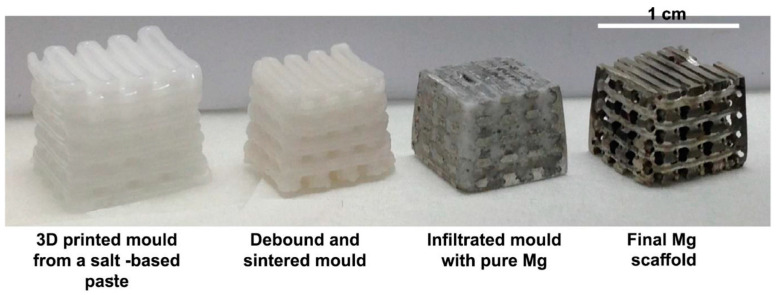
Macrographs of the process flow for the indirect additive manufacturing of a Mg scaffold at different stages, which include fabricating a NaCl mold via direct ink writing, sintering the mold, infiltrating the mold with liquid Mg, and leaching the mold to obtain the Mg scaffold. Adapted from [157].

The process of indirect AM for Mg alloys begins with creating a computer-aided design (CAD) model to define the desired architecture. Subsequently, a polymeric template is generated utilizing additive manufacturing techniques. This template is infiltrated with NaCl paste and subjected to a heating process to remove the polymer, resulting in the formation of a negative NaCl template after sintering. The Mg melt is cast into the template, and upon the dissolution of the NaCl, the Mg scaffold is fabricated. Molds are typically crafted using methods such as stereolithography or inkjet printing, utilizing building components dissolved in non-toxic organic solvents (Figure 16 and Figure 17) [62,151,155,156].

Previous research has predominantly concentrated on the I-AM of Mg alloys utilizing NaCl molds. One approach entailed infusing NaCl paste into an additively manufactured polymeric template and eliminating the polymer to yield a NaCl mold. Another technique involved direct ink writing, an extrusion-based AM method, to directly fashion the NaCl mold. These molds underwent sintering at approximately 690 °C to ensure mechanical robustness before being infiltrated with liquid Mg at around 700 °C. Eventually, the NaCl molds were dissolved by leaching in an aqueous NaOH solution [23,150,157].

The initial breakthrough in indirect 3D AM using pure magnesium occurred in 2010 [150]. However, research efforts have been sparse, with only a handful of studies exploring this technique. The main emphasis has been on the production of structured porous Mg scaffolds and the evaluation of their properties using pure Mg. Much of the research has revolved around identifying the ideal processing parameters for mold creation and the subsequent infiltration of molten Mg into the molds via pressure- or vacuum-assisted casting techniques [23].

Based on their research [20,157], it has been determined by investigators that the dimensional precision of Mg scaffolds directly printed using NaCl templates is predominantly influenced by the accuracy of the NaCl templates, as opposed to the infiltration properties of the Mg melt. Specifically, Staiger et al. [150] achieved a dimensional accuracy ranging from 88% to 95% in their successful fabrication of porous Mg scaffolds [157].

As mentioned before, I-AM is a method specifically applied for the creation of Mg scaffolds. A notable limitation of I-AM lies in the susceptibility of NaCl molds to corrosion given chloride’s role as a key factor in Mg corrosion, thereby constraining its widespread utilization. While infiltration techniques offer scaffolds with commendable macro-scale dimensional accuracy, they may not be optimal for applications necessitating open porous materials with micro-scale pores [156]. Nonetheless, the I-AM methodology still holds significant promise owing to its rapid build rates, scalability, and industrial applicability. To further harness its potential, integrating indirect AM of Mg with subtractive manufacturing techniques could yield a hybrid AM solution for crafting intricate geometries with precision [23,157].

In the future development of magnesium alloy additive manufacturing, I-AM technology has potential application prospects, which may be used to manufacture composite materials, achieve surface modification, create porous structure, or prepare functional gradient materials by combining with other additive manufacturing processes so as to further enhance the performance and scope of application of magnesium alloy parts and to meet the demand for high-performance and multi-functional materials in the fields of aerospace, automotive manufacturing, and biomedicine. 

## 3. Conclusions

In summary, modern manufacturing techniques, such as AM and hybrid AM, have become the focus of Mg alloy deposition research. The demand for magnesium and magnesium alloys is growing in industries such as aerospace, automotive manufacturing, and biomedicine. However, the deposition of a Mg alloy using AM processes faces several challenges, including evaporation, oxidation, residual stresses, thermal stress, porosity, spattering, grain growth, thermal cracking, and distortion. Some of the current problems or deficiencies require us to conduct more in-depth research to find the root cause of the problem so that we can take measures to improve or avoid the recurrence of similar problems.

In the field of magnesium alloy additive manufacturing, LPBF and WAAM, as the two mainstream technologies, each show unique advantages. LPBF technology has occupied a place in the field of precision manufacturing by virtue of its high-precision and high-performance component manufacturing capability, but the low deposition rate and limited build chamber size restrict its application in large magnesium components. In contrast, WAAM technology is known for its low cost and high efficiency and is particularly good at building large and complex magnesium alloy components, which meet the industry’s dual needs for scale and efficiency. In addition, BJ allows for the precise control of material deposition to produce customized magnesium alloy parts with complex geometries and excellent mechanical properties while being environmentally friendly and reducing waste and emissions. At the same time, FSAM can achieve grain sizes comparable to the LPBF of magnesium alloys, and coupled with its unique property of machining without melting, it demonstrates the potential to manufacture precision products. It has become a research hotspot in the field of magnesium alloy additive manufacturing due to its high efficiency, low defect rate, and suitability for the manufacturing of large components. Compared with the above AM technologies, I-AM is less popular. However, due to its high build rate, scalability, and industrial adaptability, the I-AM method still has great potential. This technique is not studied in this paper since most of the research on this technique was conducted before 2015. In the last five years, very little research has been conducted on electron beam melting (EBM) methods. Recent explorations of EBM methods have focussed on their effects on the surface properties of magnesium alloys, particularly in terms of improving corrosion resistance. However, EBM faces challenges due to electron beam dispersion caused by excessive evaporation of Mg in the vacuum chamber.

The future application of the additive manufacturing of magnesium alloy will also face many challenges, and process stability is still a key factor restricting the wide application of AM of magnesium alloy. Second, magnesium alloys are prone to metallurgical defects, such as porosity and cracks during the additive manufacturing process, which seriously affect the performance and reliability of the parts. At present, the magnesium alloy material system suitable for additive manufacturing is not yet abundant, and most of the materials are designed for traditional processes, making it difficult to give full play to the advantages of additive manufacturing. In practical applications, although additive manufacturing has advantages in customized and small batch production, its cost is still higher than that of traditional processes in large-scale production. In view of the above challenges, future research on magnesium alloy additive manufacturing should focus on the following aspects: first, deepen the understanding of the physical and chemical mechanisms of the additive manufacturing process; second, develop high-performance magnesium alloy material systems suitable for additive manufacturing; third, optimize the process parameters and process flow to improve the forming accuracy and stability; fourth, explore the multi-material composite and functional gradient material preparation technology; fifth, strengthen the cost control and production efficiency improvement research and the control and production efficiency enhancement research.

Through improvements in digital production processes; the promotion of intelligent manufacturing, data-driven decision-making; the integration of global supply chains; and the enhancement of innovation capabilities, computer–Internet technology has enabled more efficient, flexible, and intelligent production methods in the manufacturing industry. Additionally, machine learning and process modeling techniques are valuable tools, providing insights into operational mechanisms and enabling cost-effective process improvements. In conclusion, there is significant potential for integrating modern technologies to enhance the performance of Mg alloy additive manufacturing products. However, further research and innovation are necessary to overcome existing challenges and optimize process efficiency.

## Abbreviations

Abbreviations	Full Name
Mg AM	magnesium alloy additive manufacturing
SLM/LPBF	selective laser melting/laser powder bed fusion
WAAM	wire arc additive manufacturing
BJ	jetting technologies
FSAM	friction stir additive manufacturing
I-AM	indirect additive manufacturing
DOE	design of experiments
AZ31	Mg-Al-Zn alloy
Ev	energy density
DLD	direct laser deposition
RD	Relative density
MBG	Mesoporous Bioactive Glass
ZK60	Mg-Zn-Zr alloy
WE43	Mg-Al-Zn-RE alloy
AZ61	Mg-Al-Zn alloy
AZ91	Mg-Al-Zn alloy
AZ91D	Mg-Al-Zn alloy
ZK30	Mg-Zn-Zr alloy
DED	direct energy deposition
TIG	tungsten inert gas
PAW	plasma arc welding
MIG	metal inert gas
CMT(GMAW)	cold metal transfer(gas shielded metal arc welding)
GTAW	gas tungsten arc welding
AZ-series Mg-alloys	Mg-Al-Zn alloy
GW63K	Mg-Gd-Y alloy
TMAZ	Thermo-Mechanically Affected Zone
CIJ	both continuous inkjet
DOD	drop-on-demand
CAD	computer-aided design
EBM	electron beam melting
